# Editorial: Animal Welfare Assessment: Edition 1

**DOI:** 10.3389/fvets.2021.653422

**Published:** 2021-04-30

**Authors:** Edward Narayan, Alan McElligott, Alan Tilbrook

**Affiliations:** ^1^School of Agriculture and Food Sciences, Faculty of Science, The University of Queensland, St Lucia, QLD, Australia; ^2^Centre for Animal Science, Queensland Alliance for Agriculture & Food Innovation, The University of Queensland, St Lucia, QLD, Australia; ^3^Jockey Club College of Veterinary Medicine and Life Sciences, City University of Hong Kong, Hong Kong, China

**Keywords:** stress, animal welfare, livestock, wildlife, domesticated animals, biomarkers, climate change, affective state

Animal welfare refers to the well-being of the animal, and is the state of an animal as it tries to deal with the environment it is in. One way to assess the welfare state of the animal is through assessing whether it is under stress, which is the biological reaction when the animal is facing a potential threat to its welfare.

Although improvements in welfare such as through fine-scale adjustments to the animal's phenotype and its environment (e.g., genetics, husbandry, and nutrition) can improve the health of animals, good welfare does not always equate to increased productivity and vice-versa. The contextual nature of animal welfare issues transcend across different animal production systems whereby animals are managed by humans and require certain level of monitoring and care such as livestock, zoo animals, and pets. Productivity may be defined as a level of improved performance or fitness or a quantified production trait of an animal. For a farm animal, productivity could be a measured trait like meat production or milk quality while productivity for a pet species or zoo animal may not be as big a priority compared to animal welfare unless these animals are kept under breeding programs. Therefore, the current fragile atmosphere of shifting perspectives in the animal production sector and societal awareness in relation to the humane treatment of animals and use of animals for production has placed increasing pressure on finding a balance between management practices that can reduce stress, improve welfare and, equally, improve animal productivity.

In Edition 1 of this *Topic*, we show a collection of 12 peer reviewed articles which highlight the physiological, behavioral and physical health, and welfare evaluation of livestock and companion animals. It includes works of animal welfare experts, veterinarians, animal physiologists, and animal managers that will generate a healthy discussion and showcase latest studies working toward finding the harmony between animal production and welfare.

The papers presented in this special issue present new ideas and trialed research to boost animal health and welfare evaluation within intensive and extensive production systems as well as in pets and exotic species with examples from around the globe. For example, the first publication presented the physical and behavioral health indicators of cull cows in livestock markets. Sánchez-Hidalgo et al. developed a behavioral event index (BEI) comprising of cattle behaviors in the markets. Cow handler behavior was determined *via* negative tactile interactions (NTI) and the calculated index was termed as NTII. Researchers also evaluated the health status of each cattle. The researchers were successfully able to apply the cow and human related indices to determine the welfare of cull cows at livestock markets.

In the second animal welfare protocol-based research, Dalmau et al. presented a points-based animal welfare protocol for the farmed rabbits in Spanish farms by applying a multidimensional approach containing key animal-based indicators across age groups of rabbits.

In another of the works in this *Topic*, Teixeira et al., investigating animal-based welfare outcomes for pigs (*n* = 54 batches, 8,843 pigs) on-farm and abattoir in Chile, demonstrate that animal based physical health indicators can vary on farm and abattoir thus making it important to assess both individual farm and abattoir to enable through evaluation of pig welfare across the supply chain.

Tail biting is a significant welfare issue in intensive pig production. Haigh et al. applied open field and novel object test to test the animal related variation in stress responses to tail biting whereby preliminary results suggest that the differences in coping toward stress could be related to an individual pig's personality associated with either being a bold and tail biter or shy and victim.

Other contributions make relevant insights in applications of innovative tools for the animal welfare assessment. Magrin et al. studied a total of 2,161 animals from 80 Italian commercial farms. Researchers found specific lesions that could be input to develop a benchmarking system for evaluating animal health on-farm and applying this tool to improve the health and welfare of beef cattle.

In another paper, Kearton et al. show the application of associative learning behavior in Merino sheep to successfully train animals using classical conditioning to reduce contact with the aversive component of a virtual fence.

Three papers in this *Topic* focussed on companion animals, specifically dogs and cats. Davies, Scott et al., in their study successfully demonstrate the application of web based early warning system for providing 24/7 remote monitoring of dog well-being throughout the pet's lifetime. In the second dog-based paper, Clark et al. show the relationship between behavior and acute stress responses of both therapy dogs and their handlers. Salivary cortisol could be used to index stress levels of therapy dogs and applied in combination with behavior assessment to monitor the welfare of dogs. The paper by Davies, Reid, et al. was based on feline health evaluation using an online tool (HRQL). This tool helps to evaluate the impact of disease and clinical treatment on cat well-being and also supports clinical decisions and trials.

Production birds were also covered in this *Topic* with two papers. The team of researchers from Germany (Stracke et al.) inspected footpad dermatitis (FPD) in turkeys and show that improvements can be made to the current scoring system as a welfare tool through the evaluation of alterations on digits and using the total foot as a reference. In the second paper, Olschewsky et al. show the possibility of rearing slow growing turkey lines using organic husbandry which tends to improve health and welfare.

Finally, there is also a paper on fish welfare. Pedrazzani et al. show the application of an on-farm welfare assessment protocol for strengthening the practical application of on-farm welfare assessment in fish through the identification of critical welfare points.

Collectively, the *Topic* highlights current research areas and future directions in the dynamic field of animal welfare assessment. The variety of research papers demonstrate the availability of powerful tools in animal production systems through the combination of physiology, health and behavior indices and online monitoring systems to boost animal welfare for practical applications in research, commercial, and other settings.

## Author Contributions

EN conceptualized the Research Topic and collaborated with AM and AT for the coordination of this special issue. All authors contributed to the article and approved the submitted version.

## Conflict of Interest

The authors declare that the research was conducted in the absence of any commercial or financial relationships that could be construed as a potential conflict of interest.

